# Comparative genomics of human stem cell factor (SCF)

**Published:** 2017-03

**Authors:** Moein Dehbashi, Elahe Kamali, Sadeq Vallian

**Affiliations:** Genetics Division, Department of Biology, Faculty of Science, University of Isfahan, Isfahan, Iran

**Keywords:** Stem cell factor, Comparative genomics, Chimpanzee, Neanderthal

## Abstract

Stem cell factor (SCF) is a critical protein with key roles in the cell such as hematopoiesis, gametogenesis and melanogenesis. In the present study a comparative analysis on nucleotide sequences of SCF was performed in *Humanoids* using bioinformatics tools including NCBI-BLAST, MEGA6, and JBrowse. Our analysis of nucleotide sequences to find closely evolved organisms with high similarity by NCBI-BLAST tools and MEGA6 showed that human and Chimpanzee (*Pan troglodytes*) were placed into the same cluster. By using JBrowse, we found that SCF in Neanderthal had a single copy number similar to modern human and partly conserved nucleotide sequences. Together, the results approved the gene flow and genetics similarity of SCF among human and *P. troglodytes*. This may suggest that during evolution, SCF gene transferred partly intact either on the basis of sequence or function from the same ancestors to *P. troglodytes*, the ancient human like Neanderthal, and then to the modern human.

## INTRODUCTION

The c-kit ligand, also called mast cell growth factor, kit ligand (KITLG), steel factor and stem cell factor (SCF), is a growth factor playing a critical role in hematopoiesis and the generation of melanocytes and germ cells [1]. SCF also serves an important role in the development of the interstitial cells of Cajal in the intestine and the learning functions in the hippocampal region of the brain [2]. The Kit proto-oncogene (c-kit or kit) is the receptor for SCF, which is involved in juxta-membrane signaling [2]. In mice, the c-Kit gene encoding SCF is located in the white spotting (W) locus, while in human in Steel locus (Sl) on chromosome 12 and in mouse on chromosome 10 [3]. Although the absence of either SCF or c-Kit is lethal in utero, reductions in functional receptor, or ligand, could lead to aberrations in hematopoiesis, pigmentation (melanogenesis) and reproduction (gametogenesis) [2, 4].

**Figure 1:**
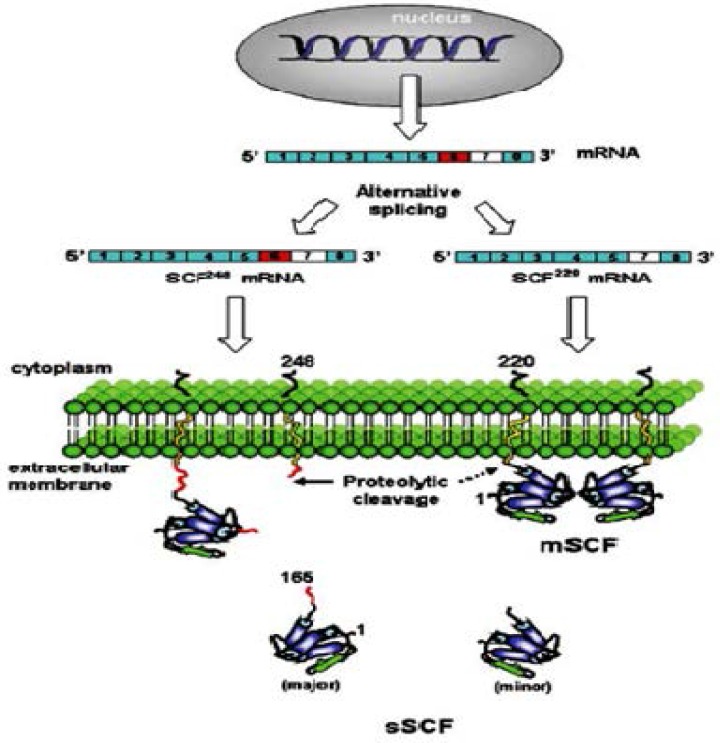
Alternative splicing of SCF. Alternative splicing of the sixth exon of SCF mRNA produces two membrane bound forms, SCF^248^ and SCF^220^. SCF248 is cleaved by proteases in the domain encoded by exon 6 ( ) to produce a soluble 165 amino acid protein (SCF165 or sSCF). SCF220 or mSCF remains membrane-bound as it lacks the proteolytic cleavage site encoded by exon 6, but it may also be shed in the region encoded by exon 7 to produce a soluble form [10].

X-ray crystallography showed that SCF is a non-covalent homodimer composed of two slightly wedged protomers, where each protomer demonstrates an anti-parallel four helix bundle fold (characteristic cytokine topology) [5]. Dimerization is done by polar and non-polar interactions between the two protomers with a large buried surface area [5]. SCF is produced by 9 exons in human, mouse and rat [6], and is present as both membrane bound (mSCF) and soluble (sSCF) forms [3]. The first SCF isoform is a 45 kDa, 248 amino acid (aa) glycoprotein (SCF^248^) localized at the cell membrane and cleaved by proteases to generate 31 kDa, 163 aa soluble protein (sSCF or SCF^163^). The cleavage site containing Val–Ala–Ala–Ser, aa 163-166 is encoded by exon 6. The alternate splicing leads to generation of the second SCF isoform around exon 6 [7]. This isoform is 32 kDa, 220 aa glycoprotein lacking exon 6 and remains membrane bound (mSCF or SCF^220^), but it may also be cleaved by proteases to generate a soluble form [8-10]. The secondary cleavage site used to produce the soluble form is located in exon 7 in mouse and used in the absence of primary cleavage site (Fig. 1).

Human SCF is translated with a 25 aa leader sequence followed by a 185 aa extracellular sequence, a 27 aa transmembrane region and a 30 aa intracellular region [11]. The N-terminal 141 residues of SCF, SCF1-141, have been recognized as a functional core which includes the dimer interface and portions that bind themselves to Kit [11]. The SCF binding to kit induces a rapid and complete receptor dimerization, leading to activation (by autophosphorylation) of the catalytic tyrosine kinase for signal transduction (1,12). In addition, a role for SCF in oogenesis and folliculogenesis has been reported [2]. Experiments on knock-in mice have demonstrated that SCF is required for spermatogonial function and also, Leydig cells, both of which express c-Kit in the testis [13]. Earlier studies had shown that SCF could enhance testosterone production of Leydig cells *in vitro*. [14]. Also, Kit signaling is important for Primordial germ cell (PGC) number and their migration [13]. Kit is highly expressed in type A spermatogonia tracked by type B spermatogonia and, at lower levels, in spermatocytes [15,16]. Erythropoiesis process is moderated by a number of growth factors, among which stem cell factor (SCF) and erythropoietin (Epo) perform a nonredundant function [17]. Also, erythroid progenitors require both c-Kit and Epo-R signal transduction pathways for their proliferation, differentiation and survival [17].

**Figure 2:**
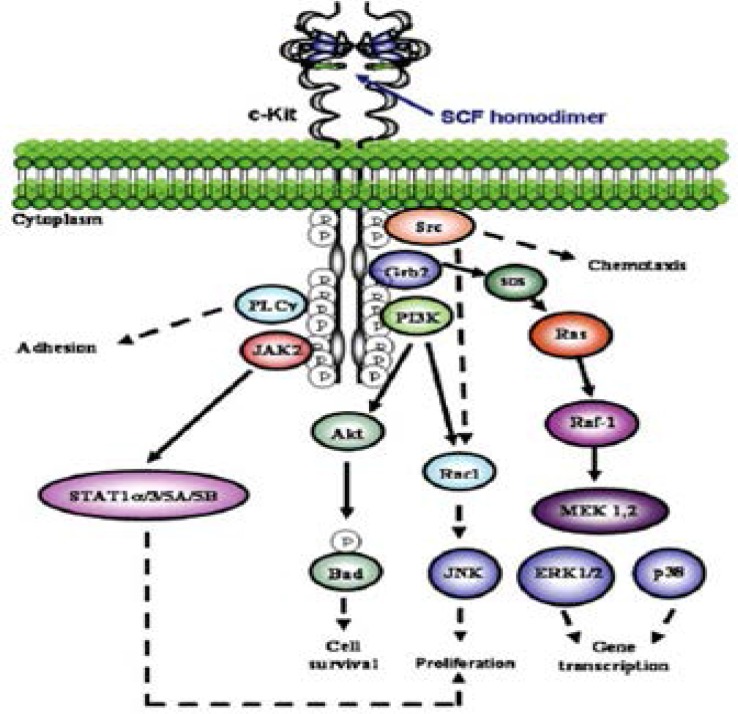
Signal transduction pathways of the c-Kit receptor. Homodimerized SCF binds itself to c-Kit, thus inducing c-Kit homodimerization and autophosphorylation, and activation of different signalling pathways. Activation of the PI3-kinase, Src and the small G protein Ras, in turn, induce the activation of different MAP-kinase pathways: ERK (extracellular-regulated kinase), p38 and JNK (Jun-N-terminal kinase). PI3-kinase also activates Akt/PKB. The Janus kinase (JAK) is associated with phosphorylated c-Kit, activatingthe transcription factor STAT (Signal Transducers and Activators of Transcription), SOS (son of sevenless); Grb-2: growth factor receptor-bound protein-2; MEK: MAP ERK kinase [10].

SCF is expressed by assorted structural and inflammatory cells in the airways and its binding to c-Kit leads to the activation of various pathways, as well as phosphatidyl-inositol-3 (PI3)-kinase, phospholipase C (PLC)-γ, Src kinase, Janus kinase (JAK)/Signal Transducers and Activators of Transcription (STAT) and mitogen activated protein (MAP) kinase pathways. SCF is a significant growth factor for mast cells, promoting their production from CD34+ progenitor cells (Fig. 2) [10].

Nowadays, there has been a growing field in the case of primate evolution, particularly *Hominoids*, in order to find the original ancestor of the human being [18]. For instance, comparative analysis of brain sizes as well as the evaluation of cognitive skills of different species, has been utilized to find out the origin of human [18-20]. Furthermore, dozens of gene sequences and other genomic markers such as retroposon insertions have largely disclosed the intertribal relationships among placental mammals [18,21]. In addition, it is obvious that phylogenomics would be a great challenging manner for re-analyzing species to establish the degrees of divergence among these great creatures [18].

Because of the significant roles of SCF, the present article, for the first time, released the results obtained from comparative genomics of human SCF. We demonstrated the evolutionary view of SCF to find the closest organism to human by orthologous SCF.

## MATERIALS AND METHODS

The sequences of human SCF (Gene ID: 4254, AC: NG_012098.1, AC: NM_000899.4; transcript variant b, AC: NM_003994.5; transcript variant a) were extracted from NCBI database. The sequences were analyzed at the levels of nucleotide and compared to all cellular life forms as well as human counterparts. The intra-species and inter-species sequences were compared to the order of primates, and the phylogenic trees were constructed for all species. 

In this study bioinformatics tools including NCBI-BLAST tool (http://blast.ncbi.nlm.nih.gov/Blast.cgi) and MEGA6 software [22] were used for sequence similarity search. Also, they were used for local alignments, *i.e.*, the maximal regions of high similarity between the query sequence and the database sequences. The sequences were searched against all reference genomic sequences, GenBank, EMBL, DDBJ, and PDB sequences. The fast nucleotide Megablast was used as the BLAST algorithm, because it could compare a query to closely related sequences, and worked best when the target percent identity was 95% or more [23]. In this way, very similar sequences were selected for alignment. In the next step, the BLAST results were used for phylogenetic tree construction by means of definite methods. In addition, fast minimum Evolution and Neighbor Joining tools were used for the evaluation of the data [24, 25]. The Maximum sequence differences of 0.75 were used and the Maximum sequence differences larger than 0.5 were considered as accurate for sequence grouping as indicated by NCBI. Also, NCBI-based algorithms including clustalW and muscle were used. 

## RESULTS

**Figure 3:**
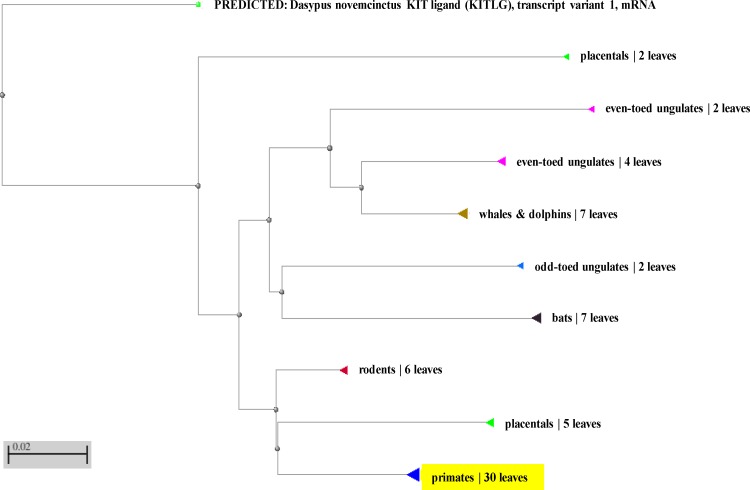
Phylogenic analysis of SCF cDNA sequences. BAST analysis and phylogenetic tree of cDNA sequences of SCF using NCBI BLAST tree view, Neighbor joining clustering method and Max Seq Difference 0.75

As the compatible results, BLAST results of the transcript variant b of human SCF (AC: NM_000899.4) by NCBI-BLAST tool (http://blast.ncbi.nlm.nih.gov/Blast.cgi) showed that human SCF was placed in primates cluster and was close to placental and far from rodents (Fig. 3). Also, we used MEGA6 software [22] to search similarity based on complete gene sequences throughout primates, showing that human SCF gene had high similarity than primates (data not shown). 

**Figure 4:**
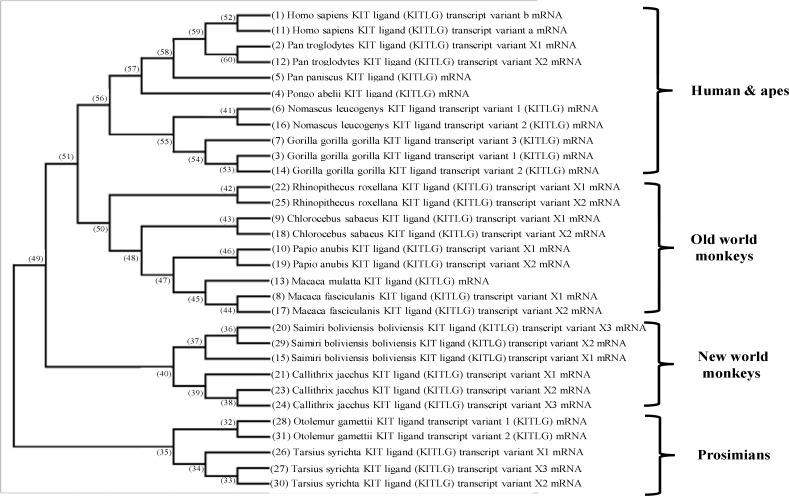
BLAST analysis of the cDNA sequence of human SCF using MEGA6 software

Among primates, BLAST analysis of the cDNA sequence of human SCF by MEGA6 software [22] showed that human SCF cDNA was placed in the cluster along with *Pan troglodytes *(Fig. 4)*. *MEGA6 analysis demonstrated that human (*Homo sapiens*) and apes main cluster was close to that of old world monkeys. However, SCF cDNA of Prosimians was far from those of human and apes, old world monkeys and new world monkeys. In the analysis of primates, both cDNA of human SCF isoforms were placed in the cluster close to two SCF isoforms of common Chimpanzee (*Pan troglodytes*) and far from *P. paniscus *and *Pongo abelii* (Fig. 4). Our analysis showed that two cDNAs of human SCF isoforms were closer to those old world monkeys and far from those of new old monkeys. Whereas, there was an interesting exception for placing the two SCF isoforms of *Nomascus leucogenys* (northern white-cheeked gibbon) in a cluster along with three SCF isoforms of *Gorilla gorilla gorilla* (western lowland gorilla). We obtained the data pertaining the number of SCF isoforms from the phylogenetic tree, indicating that three primates categorized in new world monkeys, including *Tarsius syrichta *(Philippine tarsier),* Callithrix jacchus *(common marmoset) and *Saimiri boliviensis boliviensis* (bolivian squirrel monkey) had all three isoforms among primates. However, among old world monkeys, only *Gorilla gorilla gorilla *had three SCF isoforms. Detailed analysis of primates showed that humans (*Homo* genus) and Chimpanzees (*Pan* genus) originated from a common ancestor that was far from other *Hominidae* such as gorilla and orangutan (Fig. 4). 

**Figure 5:**
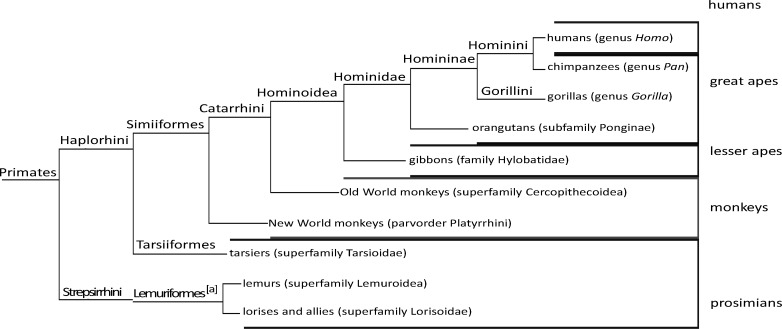
Phylogenetic tree of primates proposed by McKenna and Bell in 1997 [28].

As depicted in Figure 5, *Hominoidea* superfamily consisted of *Hylobatidae* family (*Nomascus leucogenys*, gibbon) and *Hominidae* family. Moreover, it could be expected that *Hominidae* family would be composed of *Homininae* (*Homo*, *Pan* and *Gorilla* genera) and *Ponginae* subfamilies, including orangutan (*Pongo abelii*). In addition, SCF phylogenic analysis indicated that *Pongo abelii* and *Pan paniscus *were categorized as an independent cluster in the class of humans and apes (Fig. 4). Based on our BLAST analysis, we showed that SCF during evolution had no duplication, but the variable number of isoforms among some primate might be obtained from alternative splicing variations. Moreover, when our results with NCBI-based algorithm were compared with clustalW and muscle algorithms of MEGA6, the similar data for nucleotide sequences of SCF was obtained (data not shown) [22].

**Figure 6:**
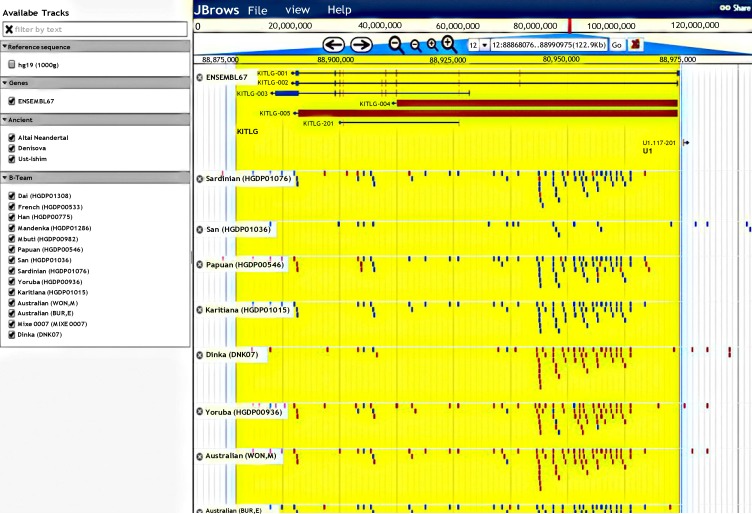
Conservation of SCF gene. The single copy and conservation of SCF gene across Neanderthal genomes using Neanderthal genomes database (https://bioinf.eva.mpg.de/jbrowse/). Blue and red dots refer to homozygous and heterozygous variants, respectively

In addition, JBrowse (https://bioinf.eva.mpg.de/jbrowse/) was utilized to survey conservation and copy number gene of SCF gene in Neanderthal genome [39]. In all Neanderthal genomes sequenced, we found partly conservation and single copy number of SCF similar to human, *Pan *and other primates*. *Our analysis showed that there were only some single nucleotide variants (SNVs) throughout SCF gene across Neanderthal genomes (Fig. 6). 

## DISCUSSION

It is assumed that the order of primates consists of the clade *Euarchontoglires,* which is placed within the clade *Eutheria* of mammalian class. From molecular evolutionary approach, *Colugos* (placental mammals) are more closely connected to primates [26]. This classification approved previous systematic studies and was consistent with the results obtained by Thorington and Anderson in 1984 [27] and McKenna and Bell in 1997 [28] (Fig. 5). These results were consistent with the currently accepted evolutionary relationships of the *Hominoidea *[29]. Traditionally, apes have been divided into the lesser apes (*Hylobatidae* family) and the great apes (*Pan*, *Gorilla* and *Pongo* genera) [18, 30]. The classification of *Strepsirrhini* and *Haplorhini* suborders of primates was introduced by Thorington and Anderson [27], and subsequently pursued by McKenna and Bell in 1997 (Fig. 5) [28, 31, 32]. According to this hypothesis, the Primates were divided into two superfamily: *Prosimii *and *Anthropoidea *[33]. *Prosimii* included all *Prosimians*: *Strepsirrhini* plus *Tarsiers* and *Anthropoidea* contained all simians [33]. In addition, modern monophyletic classifications use groups that are monophyletic [34]. A molecular phylogeny analysis by Springer and colleagues (2012) for 70 primate genera and 367 primate species was conducted based on a concatenation of 69 nuclear gene segments and ten mitochondrial gene sequences, most of which were extracted from GenBank [35]. It was suggested that living primates shared a common ancestor 71–63 Ma, and that divergences within both *Strepsirrhini* and *Haplorhini *were entirely post-Cretaceous [35]. In the present study, our results were found to be consistent with those obtained by Springer and colleagues (2012) in placing human close to *Pan* based on the genetic relationship in the clade of *Hominidae *[34]. In addition, cDNA BLAST analysis showed that some primates possessed copy number change of Isoforms. The majority of protein-coding genes have 1:1 homologues among humans, the great apes and old world monkeys sequenced so far, but gene content was not identical among primate species [36].

Particular gene families have been expanded or contracted in individual lineages. For example, 1,358 genes were identified as new duplications in the *Rhesus macaque* genome and compared with the human genome [36]. For instance, the major histocompatibility complex (MHC) gene cluster, which is critical for response to pathogens and other immunological processes, has been expanded in macaques, relative to humans [36]. However, interestingly changes in genes encoding zinc-finger transcription factors, which show gains and losses has greatly distinguished the genomes of humans, Chimpanzees and Orangutans, as well as the marked expansion of genes encoding proteins with DUF1220 domains in humans. This might be related to the expansion of human brain size [36].

Nevertheless, the draft quality of current non-human primate genome assemblies makes it difficult to define all copy-number variations accurately [36]. One can compare gene lists from different assemblies, but gaps and other issues in these assemblies create ambiguity [36]. The present data suggested that human and Chimpanzees underwent more rapid changes in gene copy number than Orangutans and rhesus macaques. Among the great apes, gorillas showed more copy-number variants than others [36]. However, complete analyses await additional data, including better genome assemblies and information concerning copy-number polymorphism in non-human primates [36]. Segmental duplications (that is, chromosomal regions >1 kb, that are >90% identical to other segments in the same genome) are a significant aspect of primate genome structure and dynamics. Duplication and deletion of these segments are active in the human genome [36]. Some of these mutations are apparently neutral, but many may lead to adverse consequences and diseases. Similar to the way in which segmental duplications create variation among humans, these duplications are ‘drivers’ of evolutionary change across primate genomes. About 5% of the human and Chimpanzee genomes, and 3.8% of the orangutan genome, comprise segmental duplications [36]. The human and great ape genomes are enriched with dispersed duplications, as they have been subjected to an interval after their divergence from old world monkeys, when the production of new duplications was particularly active [36]. Many expansions of specific protein-coding gene families result from segmental duplications, which sometimes involve repeated expansions of a given sequence [36]. Some genes within segmental duplications show evidence of positive selection on both protein-coding sequence and copy number [36]. Among the great apes, some of these expansion events have occurred as independent parallel events in different lineages strengthening the interpretation that these genomic changes were often the result of positive selection on both gene copy number and nucleotide sequences [36-38]. Generally, by utilizing comparative genomics at the level of nucleotide sequences, we obtained novel data regarding SCF key gene and explained the genetic relation between modern human, Neanderthal and primates. All results approved the gene flow and genetics similarity of SCF among human and *P. troglodytes *[40]. It seems that during the evolution, SCF gene, as a key gene, was transferred partly intact on the basis of nucleotide sequences from the same ancestors to *P. troglodytes*, the ancient human like Neanderthal, and then modern human. 
